# Ultrafast dynamics and ablation mechanism in femtosecond laser irradiated Au/Ti bilayer systems

**DOI:** 10.1515/nanoph-2023-0497

**Published:** 2023-11-30

**Authors:** Yiling Lian, Lan Jiang, Jingya Sun, Wenpan Tao, Zhicheng Chen, Gen Lin, Ziqian Ning, Manlou Ye

**Affiliations:** Laser Micro/Nano Fabrication Laboratory, School of Mechanical Engineering, Beijing Institute of Technology, Beijing 100081, P.R. China; Yangtze Delta Region Academy of Beijing Institute of Technology, Jiaxing 314019, P.R. China; Beijing Institute of Technology Chongqing Innovation Center, Chongqing 401120, P.R. China

**Keywords:** opto-thermo-mechanical coupling, pump probe imaging, MD-TTM, phase transition

## Abstract

The significance of ultrafast laser-induced energy and mass transfer at interfaces has been growing in the field of nanoscience and technology. Nevertheless, the complexity arising from non-linear and non-equilibrium optical-thermal-mechanical interactions results in intricate transitional behaviors. This complexity presents challenges when attempting to analyze these phenomena exclusively through modeling or experimentation. In this study, we conduct time-resolved reflective pump-probe imaging and molecular-dynamics coupled two-temperature model (MD-TTM) simulations to investigate the ultrafast dynamics and ablation mechanism of Au/Ti bilayer systems. The calculated energy absorption curves indicate that Au film reduces the energy deposition in the underlying Ti layer, resulting in reduced melting and evaporation rate of Ti. The phase transition process induces different mechanical responses. The potential energy patterns indicate that the expansion of vapor Ti extrudes the surface Au layer outward. In simulated stress distribution images, the Au layer can hamper the expansion of the vapor-phase Ti and brings dynamic compressive stress to the residual Ti layer. When the compressive stress transforms into tensile stress, the material is removed through mechanical damage. Therefore, both Au and Ti in the 20 nm Au-covered Ti are completely removed. Our approach elucidates the ablation mechanism within the Au/Ti bilayer system and offers fresh insights into managing thermo-mechanical responses within analogous systems.

## 1 Introduction

The femtosecond laser has gained considerable attention for its ability to achieve ultra-high precision processing [1, 2] in various systems, such as semiconductors [3, 4], flexible displays [5, 6], and super-hard materials [7–9], owing to its ultrahigh peak power and ultrashort pulse duration. However, femtosecond laser processing involves numerous non-equilibrium phenomena 10–14]. For instance, the pulse duration is shorter than the electron-lattice equilibrium time results in an imbalance in thermal [15–17] and stress distribution [18, 19] within the irradiated area. Elevated temperatures and significant mechanical stress can both lead to material damage and the creation of various micro and nanostructures [20–23]. Understanding these processes becomes crucial for enhancing manufacturing capabilities, as thermal and mechanical damage coexist and interact [24–27], making it challenging to comprehend their intricacies.

Considerable efforts have been dedicated to studying the thermo-mechanical processes of ultrafast laser ablation. The two-temperature model [28, 29], based on the continuum medium theory, is utilized to describe the evolution of ultrafast electron-lattice temperature at different locations. At low excitation fluence, parameters such as electron heat capacity and electron thermal conductivity often exhibit linear relationship with temperature ranging, while electron–phonon coupling coefficient shows a constant value to temperature, enabling accurate validation of simulation results through experiments. For instance, Guo et al. [30–32] employed the two-temperature model (TTM) to calculate instantaneous temperature changes on the surface of a gold (Au) film, and simulated reflectance using the extended Drude model. After introducing interband electron transitions, they achieved excellent agreement at multiple probing wavelengths. With the escalation of laser fluence and the consequent occurrence of material damage and removal, the model originally designed for low-temperature ranges progressively becomes insufficient. Lin et al. [33] introduced a critical temperature criterion and dynamically eliminated model grids above the critical temperature during the simulation to predict the ablation morphology. To address the limitations of TTM in elucidating mechanical effects, a method known as molecular dynamics coupled two-temperature model (MD-TTM) has been proposed [34–37], which has established relationships between macroscopic parameters such as potential energy, stress, velocity, ablation rate to underlying atomic motion and positions. In this context, MD-TTM has found extensive application in the study of multiscale material transition dynamics. This includes simulating the distribution of solid–liquid–vapor phases in laser-irradiated Au films and predicting mechanical spallation in Ti and Ni based alloys.

Due to the different thermal [33, 38] and mechanical [39, 40] parameters on both sides of the interface, heterogeneous materials often show entirely new properties [41, 42]. Combining metals with semiconductors can trigger highly effective hot electron transfer [43, 44], making it an ideal choice for device. Au–Ti bilayer metal materials [45, 46], known for their outstanding electrical conductivity and robust bonding, are widely employed as electrodes in diverse devices. The introduction of laser processing enables the fabrication of patterns on these devices or electrodes, substantially expanding the range of applications for heterogeneous materials. But thermo-mechanical coupling processes and the related material removal mechanisms are more complex compared to homogeneous materials [47–52]. During the ablation process, the heterogeneous interfaces results in temperature jumps during laser heating and induce interfacial stress peak in materials deformation. Both thermal and mechanical factors can cause material damage and influence subsequent structural formation [17], which makes it challenging to describe the ultrafast transition process using only MD-TTM simulations. The pump-probe imaging technique allows for the measurement of transient optical properties, which can subsequently be used to determine laser-induced transitions [53]. The abruptly increased reflectivity represents a coulomb explosion, and the subsequent decrease represents a phase explosion. Even electron relaxation time, plasma expansion rate, and shockwave pressure could be resolved. Moreover, not only does the measuring timescale [54, 55] of the pump-probe imaging technique fit well with that of MD-TTM, but also the measured parameters [56, 57] are similar to that simulated by MD-TTM. In this context, the potential approach of correlating computational results with turning points or trends observed in experimental data seems promising for the study of the intricate thermo-mechanical dynamics of heterostructures [58].

In this study, we designed a bilayer system consisting of surface Au and underlying titanium (Ti) and investigated the photon energy deposition, interface thermal-mechanical coupling, and damage mechanisms. The ultrafast reflective images show a lowered melting rate of the irradiated area with increasing Au thickness, and Newton’s rings represent mechanical spallation. The simulation results indicate that the Au film reduces the energy deposition and lowers the melting and evaporation rate of the underlying Ti. The vapor phase Ti then extrudes the surface Au outward, which aligns with the observed Newton’s rings and the calculated evolution potential energy patterns. Because of the robust expansion resistance stemming from the Au surface to the vaporized Ti, there is also a development of significant compressive stress on the underlying unmelted Ti. When this compressive stress transforms into tensile stress, it enhances material removal. Consequently, the most substantial stress response occurs in the underlying Ti layer of 20 nm Au-covered Ti, resulting in a notable improvement in material removal. The approach of associating ultrafast imaging findings with theoretical simulations, as outlined in this study, carries significant implications for investigating the nanoscale dynamics of photon-thermal-mechanical transfer. It not only provides insights into the complex coupling dynamics in the Au–Ti system studied here but also holds potential applications in various other heterogeneous material systems.

## 2 Experimental section

In our experimental setup, we fabricated uniform films on the [100] plane of sapphire substrates, which are single crystals, using a magnetron sputtering process. The Ti and Au target materials employed had a purity level exceeding 99.9 %. Specifically, we prepared three samples: the first sample consisted of a 160 nm thick layer of titanium, the second sample had a 160 nm titanium layer coated with an additional 20 nm Au, and the third sample featured a 160 nm titanium layer coated with a 35 nm layer of Au. For the reflective pump-probe experiments [8, 54, 59], we employed a femtosecond laser with a pulse duration less than 140 fs (YactoCrystal-FL-20) and a central wavelength of 1030 nm. The optical setup followed previous reports [24, 59]. The femtosecond laser pulse, with a wavelength of 1030 nm, was split into two sub-pulses. The 1030 nm pump pulse was directed onto the sample surface at a 60-degree angle with respect to the sample surface and was focused using a plano-convex lens with a focal length of 45 mm. The probe pulse was subjected to frequency doubling by passing through a beta barium borate crystal, thereby generating a femtosecond laser pulse with a wavelength of 515 nm. The probe pulse was captured through a 50× objective and filtered with a bandpass filter centered at 515 nm upon entering the camera. By synchronously triggering both the camera and laser while setting the camera exposure time to 1 ms, we achieved single-pulse exposure imaging. The obtained images were processed to ascertain relative alterations in reflectivity. We investigated surface morphology by employing a Zeiss scanning electron microscope, and we utilized the energy-dispersive spectroscopy module to gauge the elemental composition of distinct regions. We employed the MD-TTM model [34, 60] for the theoretical simulations, performing calculations within a simulation range of 0 ps–72 ps. The Johnson and Christy model [61] is used to calculate the optical propagation in Au, and the CRC model [62] is used to calculate the optical propagation in Ti. The parameters used in the MD and TTM parts are all referred to in the reported results [63–66].

## 3 Results and discussions

**Figure 1:**
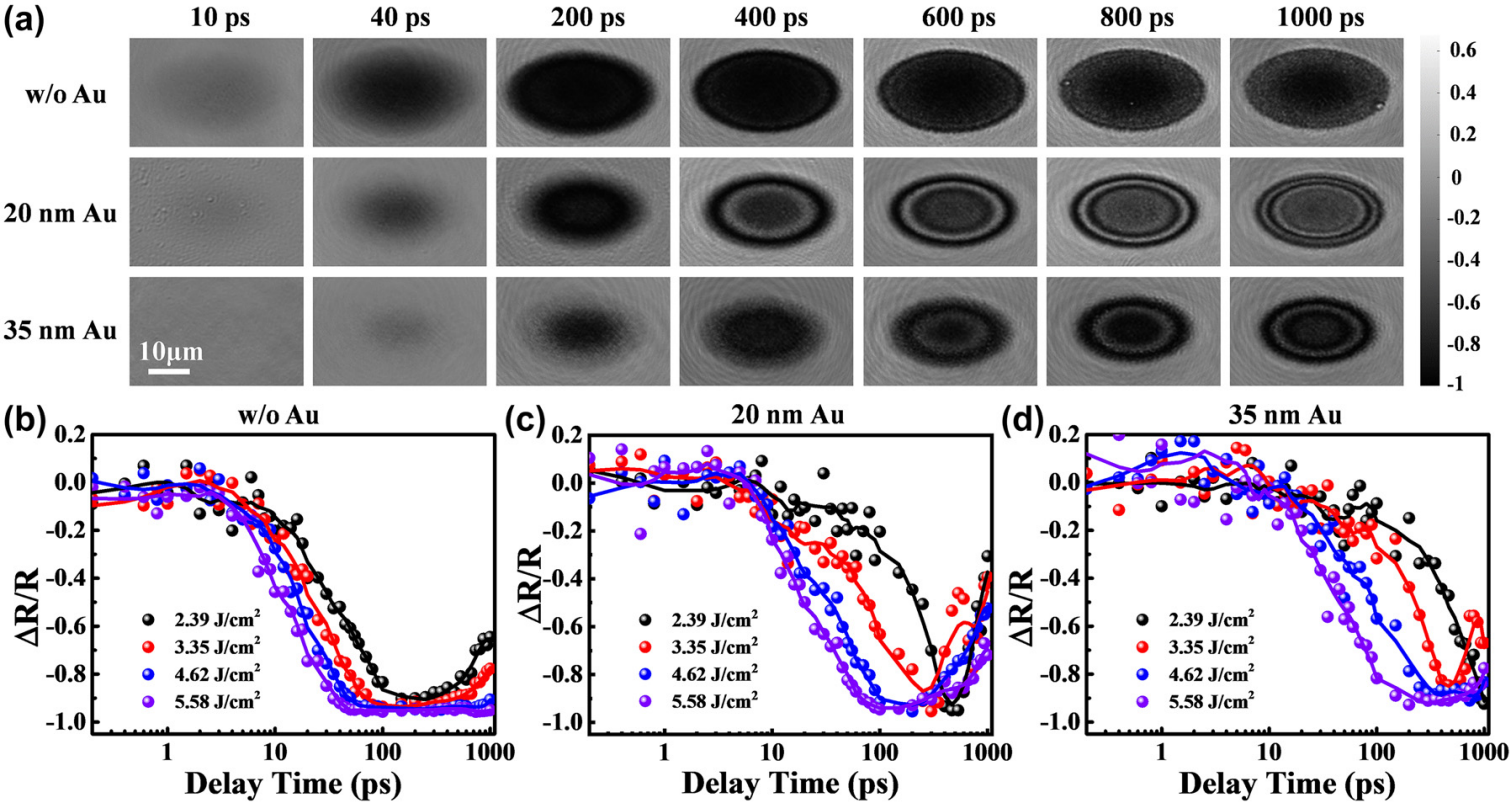
Time-resolved reflective pump-probe results. (a) Spatial-temporal images of samples without Au, with 20 nm, and 35 nm Au. The images were obtained under 4.62 J/cm^2^ irradiation. (b), (c), and (d) show the extracted relative central reflectivity of samples without Au, with 20 nm, and 35 nm Au, respectively. The original images are shown in Figure S1.

Figure 1 illustrates the dynamics of ultrafast reflectivity under irradiation with a laser fluence of 4.62 J/cm^2^. The resulting image takes on an elliptical shape as a consequence of the 60-degree incidence of the pump beam. In the scenario involving a single-layer Ti film, the region under irradiation becomes dark within the initial 10 ps, indicating the early onset of Ti film melting. With a probe delay of 40 ps, the central region expands and further darkens, indicating the ongoing melting process. Beyond 200 ps, the entire irradiation region becomes completely dark. Notably, alternating bright and dark ring patterns emerge at the irradiation edges, attributable to the refractive index difference between the ejected material and air, leading to periodic Newton’s rings as a result of interference between the probing pulse at the front and bottom of the ejecta [67]. After 800 ps, the irradiated region gradually brightens, indicating the start of the cooling process. The surface melting rate is reduced for the Ti film coated with a 20 nm Au layer. At a 40 ps delay, only the central region of the irradiation exhibits a decrease in reflectivity, while the edges remain unaffected. With a longer delay of 400 ps, the number of Newton’s rings decreases, and they appear wider width compared to the single-layer Ti case, indicating decreased ejection strength [68]. When the Au thickness is increased to 35 nm, the melting rate further slows down. The melting dynamics are analyzed based on the extracted normalized reflectivity changes shown in Figure 1(b)–(d). The dots in the figures depict the relative reflectivity values extracted from their respective delays. Given that these dots are discrete and may exhibit variations due to factors like fluctuating pulse energy and beam directionality, we have represented the scattered points at different delays under the same parameters using smoothing lines. *R* and *R*_0_ represent the intensities of a shadowgraph image with and without pump pulse irradiation, respectively. △*R* is defined as *R* − *R*_0_. In Figure 1(b), the relative central reflectivity continuously decreases, starting from approximately 3 ps for all irradiation conditions. However, the subsequent melting process is entirely dependent on the laser irradiation fluence. When the laser fluence is 2.39 J/cm^2^, it takes about 100 ps for the relative central reflectivity to decrease to approximately −0.93 at the bottom. For a laser fluence of 5.58 J/cm^2^, the time for reflectivity to decrease shortens to 40 ps. The respective times required for complete surface melting are 500 ps and 100 ps for the 20 nm Au sample, as depicted in Figures 1(c) and 1000 ps and 200 ps for the 35 nm Au sample, as shown in Figure 1(d). The significant differences in reflectivity evolution mentioned above indicate substantial variations in the material removal process.

**Figure 2:**
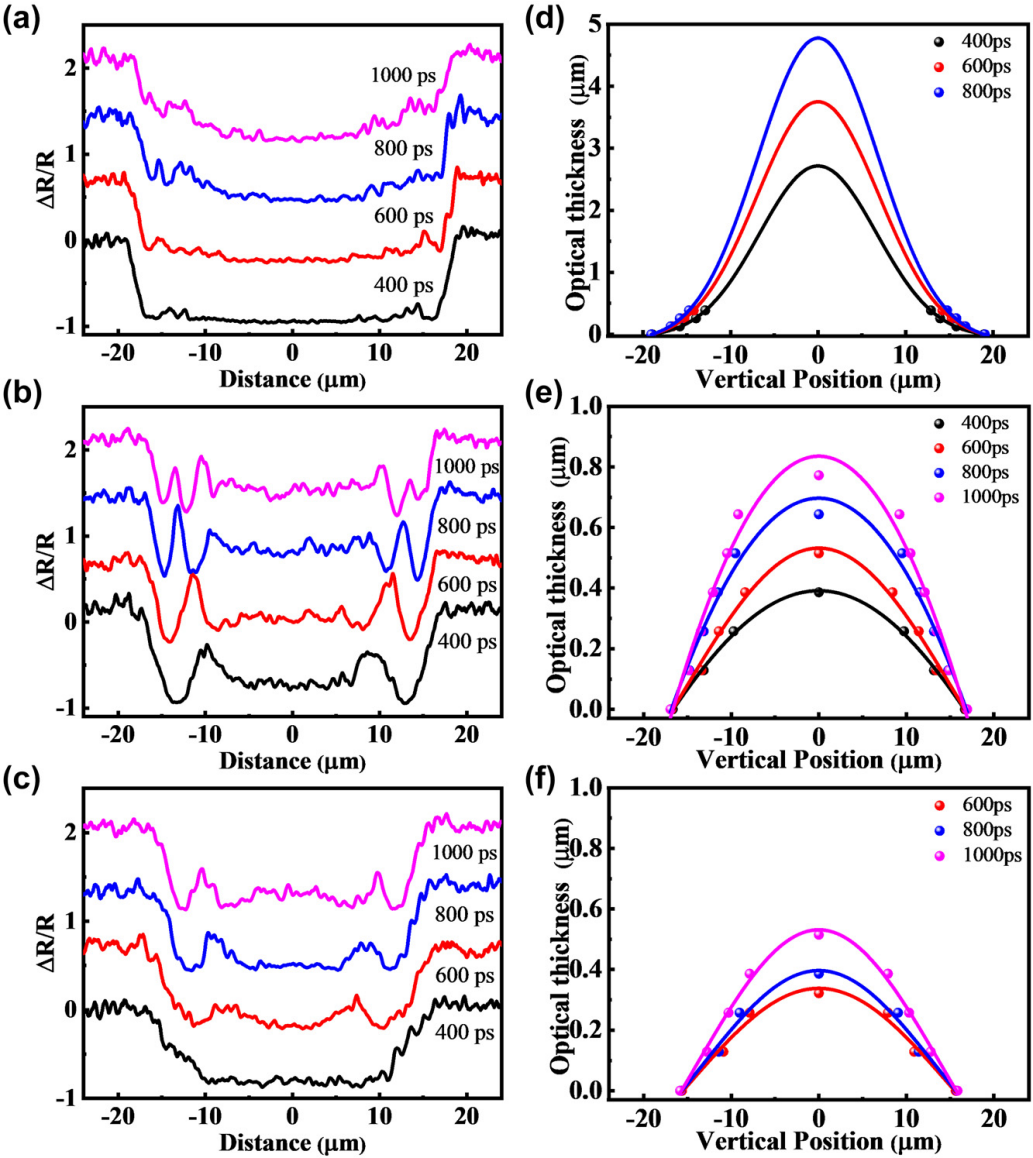
Analysis of the time-resolved images. (a)–(c) Show the cross-sectional reflectivity patterns at delays ranging from 400 to 1000 ps, with an excitation fluence of 4.62 J/cm^2^. (d) to (f) Represent the corresponding fitted optical thickness, where the vertical axis represents the actual thickness *d* from the front to the bottom multiplied by the refractive index *n* of the ejected material.

The distinction in the ablation process, characterized by solid–liquid–vapor formation and ejecta expansion [69], can be conveniently investigated by analyzing the transient Newton rings in detail. By extracting the axial spatially resolved reflectivity and identifying the maximum and minimum reflectivity values on the cross-section, the brightness variation of Newton’s rings can serve as a periodic reference for the variation in optical thickness. As per the interference law, when the optical path difference satisfies the condition 2nd = 2*k* × *λ*/2, it leads to enhanced interference, resulting in the formation of bright rings. Conversely, when the optical path difference satisfies 2nd = (2*k* + 1) × *λ*/2, it causes destructive interference, giving rise to dark rings. In this equation, *n* represents the refractive index, *d* stands for thickness, *k* denotes the number of periods or rings, and *λ* represents the wavelength of the probe beam. Within the eruption contour range, the distance continuously varies from 0 to the eruption front, creating what are known as Newton’s rings. In this context, the outermost edge of the transient reflectance image, where the dark ring appears, is defined as having an optical thickness of 0. With each additional ring moving inward, the optical thickness increases by *λ*/2. For this experiment, the probing light has a wavelength of 515 nm, and *λ*/2 is approximately 276 nm. Since the front edge of ablation exhibits a Gaussian shape in metals, semiconductors, and insulators [67], the optical thickness can be analyzed through Gaussian fitting of the bright and dark locations. The center of the Gaussian fit to the contour corresponds to the location of the eruption front. Figure 2(a)–(c) illustrate the cross-sectional reflectivity patterns at delays ranging from 400 to 1000 ps, with an excitation fluence of 4.62 J/cm^2^. On the other hand, Figure 2(d)–(f) represent the corresponding fitted optical thickness, where the optical thickness denotes the actual thickness (*d*) from the eruption front to the bottom multiplied by the refractive index (*n*) of the corresponding ejecta material. In Figure 2(a)–(c), the size of the ablation region remains unchanged in all three samples after 400 ps. However, there is a noticeable difference in the characterization of Newton’s rings. In Figure 2(a), distinct reflectivity jumps, indicating localized minimum and maximum values, are observable at the edges of the ablation region between 400 and 800 ps. However, these jumps disappear at 1000 ps. This disappearance can be attributed to two factors. Firstly, during the ablation process, the boundary between air and ejected material gradually becomes less defined, making it challenging for the conditions required for interference to be met. Secondly, as the time delay increases, the distance over which material is ejected also increases. According to the fitted curves shown in Figure 2(d), the radial distance (*d*) that satisfies the interference condition for a single Newton’s ring becomes too small to be reliably distinguished, resulting in difficulties in discerning the finer details. This corresponds to the dense fluctuations in reflectivity observed near the edges of the ablation region at 1000 ps. In the center of the ablation region, the reflectivity remains stable without spatial oscillation. This indicates that the central region is primarily influenced by thermal effects, while only the edges exhibit characteristics of mechanical damage. Based on the fitted optical thickness shown in Figure 2(e), the ejection intensity of the 20 nm Au film is significantly weakened. The entire irradiated region, from the center to the edges, displays the characteristics of Newton’s rings. Combining this with the melting rate shown in Figure 1, it can be inferred that the Au film reduces thermal damage and results in significant spallation behavior. When the thickness of the Au film is further increased to 35 nm, as shown in Figure 2(f), Newton’s rings have not yet formed at 400 ps. This indicates a substantial suppression of both thermal and mechanical transitions at this early time point.

**Figure 3:**
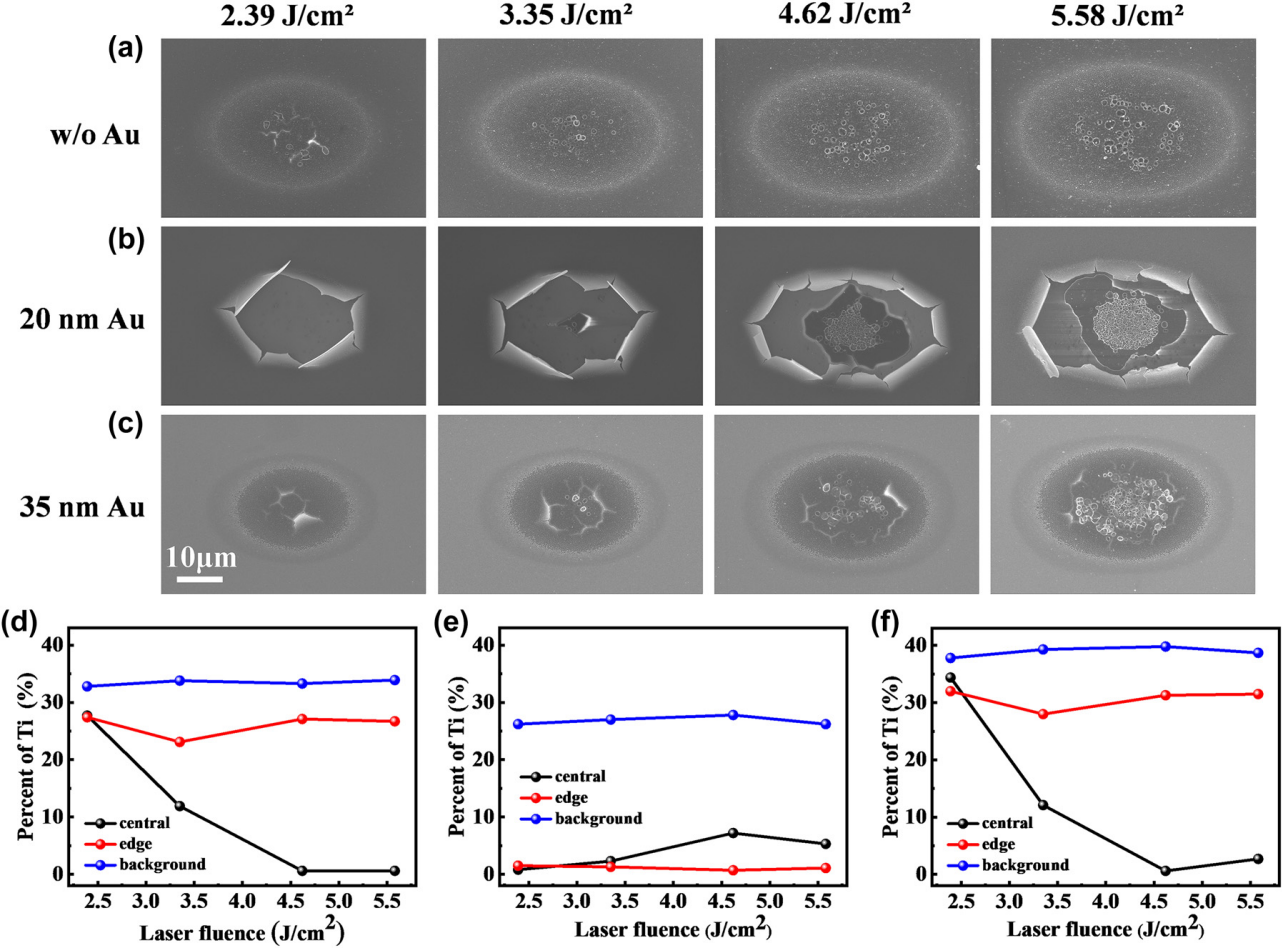
Surface morphology of the (a) single layer Ti, (b) 20 nm Au-covered Ti, and (c) 35 nm Au-covered Ti was examined under laser fluences ranging from 2.39–5.58 J/cm^2^. (d), (e), and (f) indicate the Ti content of specific regions corresponding to (a), (b), and (c), respectively.

The surface morphology depicted in Figure 3 demonstrates the substantial influence of the Au film thickness on the ablation outcomes. In Figure 3(a), a distinct elliptical ablation morphology is depicted at the edges, along with densely distributed nanoparticles within the ablation pit. At a fluence of 2.39 J/cm^2^, cracks start to appear in the central region. As the fluence increases further, circular micro-pits with diameters in the micrometer range begin to form. The size and density of these micro-pits increase as the laser fluence is increased, and when the fluence reaches 5.58 J/cm^2^, the micro-pits in the central region of irradiated area start to overlap. Previous studies suggest that this morphology is attributed to thermal effects during the ablation process. The removal of the liquid phase at the bottom leads to the formation of metal bubbles. After the rupture, the circular pit formed. The energy-dispersive spectroscopy (EDS) results in Figure 3(d) reveal a gradual decrease in Ti content in the central region as the fluence increases, while the Ti content at the edges remains relatively unchanged. Based on the aforementioned analysis, it can be concluded that in the case of a single-layer Ti film irradiated at low fluence, mechanical damage dominates, resulting in the formation of cracks. As the fluence increases, thermal effects become more pronounced, resulting in the creation of micrometer-scale micro-pits. In Figure 3(b), we observe curling and crack extension traces at the edges of the irradiated region. These phenomena can be attributed to the occurrence of ductile flow of the molten surface Au film, which aligns with earlier research findings. At a fluence of 2.39 J/cm^2^, the bottom of the ablation pit appears very smooth, as evident from the element content results in Figure 3(e), where both layers of the Au–Ti film are completely removed. As the fluence is increased to 3.35 J/cm^2^, residual Ti starts to appear in the central region. With further increases in fluence, the area of residual Ti also expands. The observed cracks at low fluence are caused by mechanical tearing, and as the fluence increases, thermal effects become more significant. The initial ejection partially obstructs subsequent ejection, leading to a decrease in Ti removal as the fluence increases. In Figure 3(c), pronounced crack curling and molten micro-pit structures are observed. According to the element content results in Figure 3(f), the observed crack curling in this case is attributed to the distortion of Ti, not Au. The overall evolutionary patterns in Figure 3(c) align with those observed in Figure 3(a) and (b). As the fluence increases, the impact of mechanical effects diminishes, while thermal effects become more pronounced.

**Figure 4:**
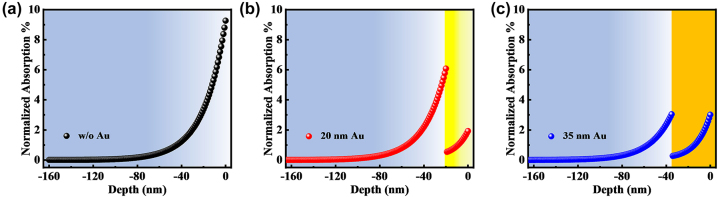
The calculated depth-dependent energy normalized absorption of (a) Ti, (b) 20 nm Au – covered Ti, and (c) 35 nm Au – covered Ti. The reference point for depth is at zero, representing the sample’s surface. Negative coordinate values correspond to various depths within the sample, indicating the normalized absorption at those specific depths.

To gain further insights into the ablation mechanism of Au–Ti systems induced by femtosecond laser, we employed MD-TTM to analyze the thermomechanical response during the material removal process. The total deposited energy is regarded as 100 %, the normalized absorption can be used to denote the fraction of energy deposited at a specific location as light traverses through material. Consequently, we employed the Finite-Difference Time-Domain (FDTD) method to compute the normalized absorption and have displayed it in Figure 4. The surface of the single-layer Ti exhibited a normalized absorption of approximately 9 %, which sharply decreased to less than 1 % within a depth of 50 nm as shown in Figure 4(a). In the 20 nm Au-coated Ti system, as illustrated in Figure 4(b), the normalized absorption at the interface between Au and Ti was below 1 %. However, it surpassed 5 % at the Ti interface. When the thickness of the Au film was increased to 35 nm (Figure 4(c)), the peak energy normalized absorption on the Au surface became comparable to that in Ti. These energy distribution characteristics significantly influenced transition behaviors, such as transient potential energy and dynamic stress.

**Figure 5:**
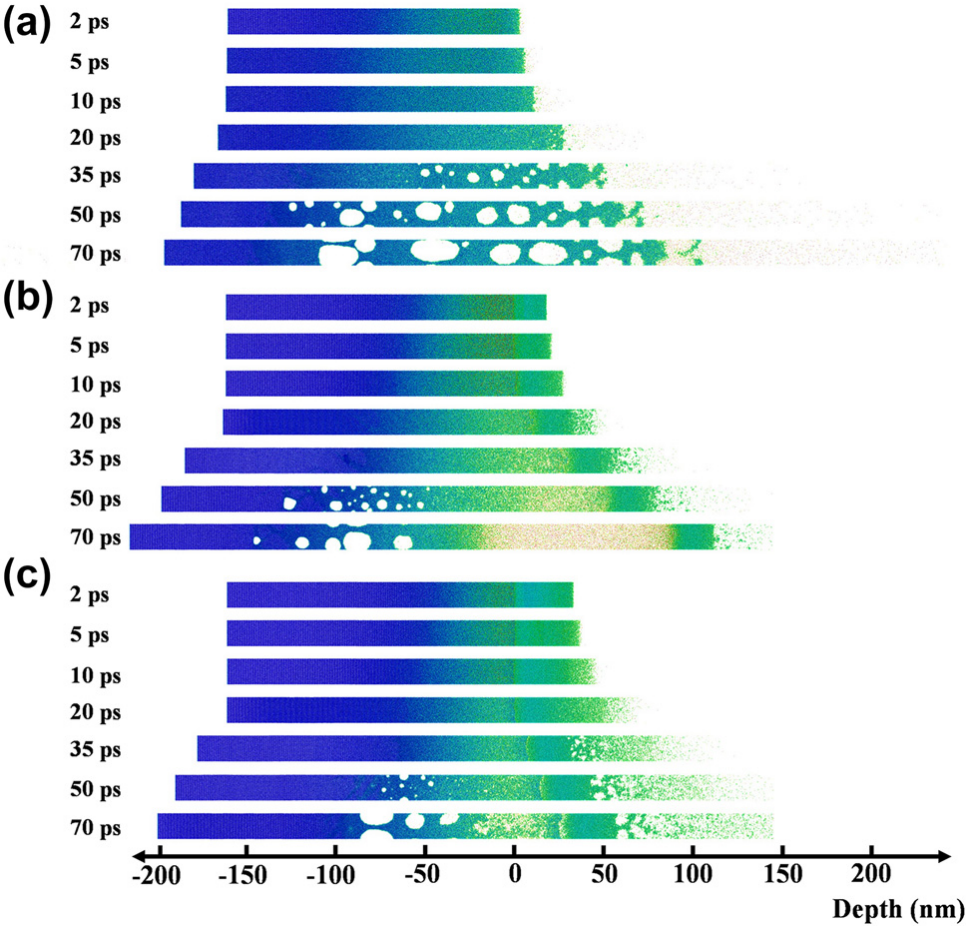
The time-resolved atomic potential energy landscapes are shown for (a) single-layer Ti, (b) 20 nm Au-covered Ti, and (c) 35 nm Au-covered Ti. The horizontal axis, in this context, represents the position of atoms within the calculation range. The zero point represents Ti surface in (a) and Au–Ti interface in (b) and (c).

Figure 5 illustrates atomic snapshots of potential energy evolution for three samples: single-layer Ti, 20 nm Au-covered Ti, and 35 nm Au-covered Ti. The simulated laser fluence used in the calculations is 4.62 J/cm^2^. In Figure 5(a), the surface atoms of the single-layer Ti rapidly melt within 2 ps and subsequently vaporize, leading to the ejection of atoms from the surface into the air. The remaining liquid Ti expands outward, giving rise to a layered vapor–liquid–solid structure. This particular structure does not possess a secondary reflecting surface for enhancing interference, which leads to the absence of Newton’s rings in the central region of the single-layer Ti, as depicted in Figure 1(a). It’s worth noting that distinct voids become visible at the bottom of the Ti layer. By 70 ps, these voids grow and coalesce with each other, potentially serving as the points of origin for the micro craters observed in Figure 3(a). However, the material removal process depicted in Figure 5(b) exhibits significant differences. The surface Au layer undergoes a transition from melting to vaporization, while the bottom atoms of the Au layer remain in a state of liquid–solid coexistence with lower potential energy. This indicates that the bottom of the Au film experiences a rise in temperature and deformation but does not completely melt. It is worth noting that in the atomic snapshot shown in Figure 5(b), the top Ti atoms gradually transform into the vapor phase, with a higher atom density compared to Figure 5(a). This is due to the limited expansion of the vapor phase by the surface Au film. Additionally, the formation of bottom cavities occurs more rapidly and results in greater depth compared to Figure 5(a), signifying stronger mechanical effects. However, due to the increased energy deposition on Au and reduced energy deposition on Ti, Figure 5(c) shows an augmented vapor phase thickness on the Au surface but a diminished thickness at the Ti interface. The growth of cavities is even suppressed. The generation and growth rules are consistent with the above results.

**Figure 6:**
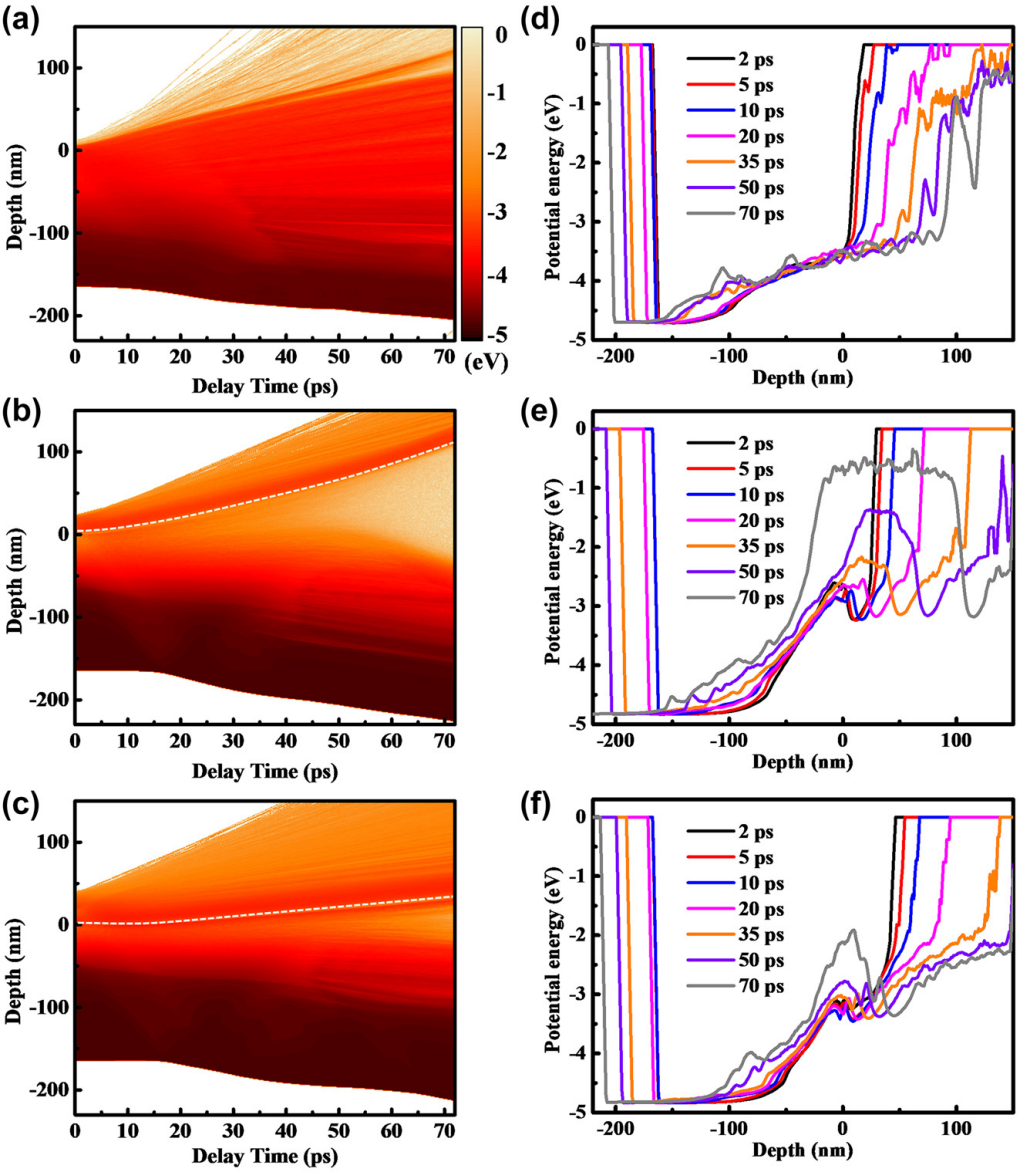
The spatio-temporal evolution of potential energy is depicted in (a), (b), and (c), which show potential energy contour maps for the single-layer Ti, 20 nm Au – covered Ti, and 35 nm Au – covered Ti samples. These patterns illustrate the evolution of stress with depth and time within 70 ps. Furthermore, Figures (d), (e), and (f) depict potential profiles at specific time delays corresponding to (a), (b), and (c), respectively. The white dotted lines denote the Au–Ti interface.

Figure 6 provides a more detailed analysis of the evolution of potential energy compared to Figure 5, enabling a precise characterization of the melting front and vapor–liquid interface based on potential energy. In Figure 6(a), a distinct boundary is observed between the vapor phase and the liquid phase. For Ti, a potential energy greater than −1 eV indicates the vapor phase, which is determined based on the distribution of atomic clusters in Figure 5. The locations where the potential energy diverges from its initial state indicate the onset of melting, leading to a mixture of solid and liquid phases. Nevertheless, the demarcation for the liquid phase is less distinct since the potential energy distribution of the liquid phase is wide, covering the spectrum from solid–liquid coexistence to the transition into the vapor phase. As a result, Figure 6(a)–(d) portray a characteristic layered structure comprising three phases: vapor, liquid, and solid. The vapor and liquid phases rapidly expand into the air, while the melting front between the solid and liquid phases gradually moves inward. In Figure 6(b)–(e), the phase transition processes of Au and Ti are presented as the surface of Ti is covered with a 20 nm Au layer. It is observed that within 2 ps, the potential energy of the surface Ti increases from −4.8 eV to −2.8 eV. However, it remains at −2.8 eV until 20 ps. This behavior is attributed to the presence of the Au layer on the surface. The slower temperature rise is due to the lower energy absorption and smaller electron–phonon coupling coefficient of the Au layer. In contrast, the underlying Ti absorbs more energy and has a larger electron–phonon coupling coefficient, resulting in faster melting. The ejection of molten Ti is impeded by the presence of the Au layer, leading to a slower change in the potential energy of Ti. As the liquid Ti expands further, the potential energy of Ti keeps rising until it reaches the vapor phase at 70 ps. In the case of the surface Au layer, the potential energy image indicates a reduction in the thickness of the Au layer with low potential energy. For instance, at a delay of 50 ps, the thickness of the Au layer with potential energy lower than −2 eV is approximately 70 nm, whereas at 70 ps, the thickness of the low-potential-energy layer is reduced to 50 nm. This indicates that the Au layer continues to melt during the expansion process. Two factors may contribute to this phenomenon: the electron-lattice coupling temperature rise of Au itself and the collisional heating from the underlying vaporous Ti. In Figure 6(c)–(f), owing to the greater absorption of laser energy by the 35 nm Au layer, a reduced amount of energy is deposited into the Ti. As a result, alongside the accelerated melting of the Au surface, the phase transition and expansion of Ti are notably impeded.

**Figure 7:**
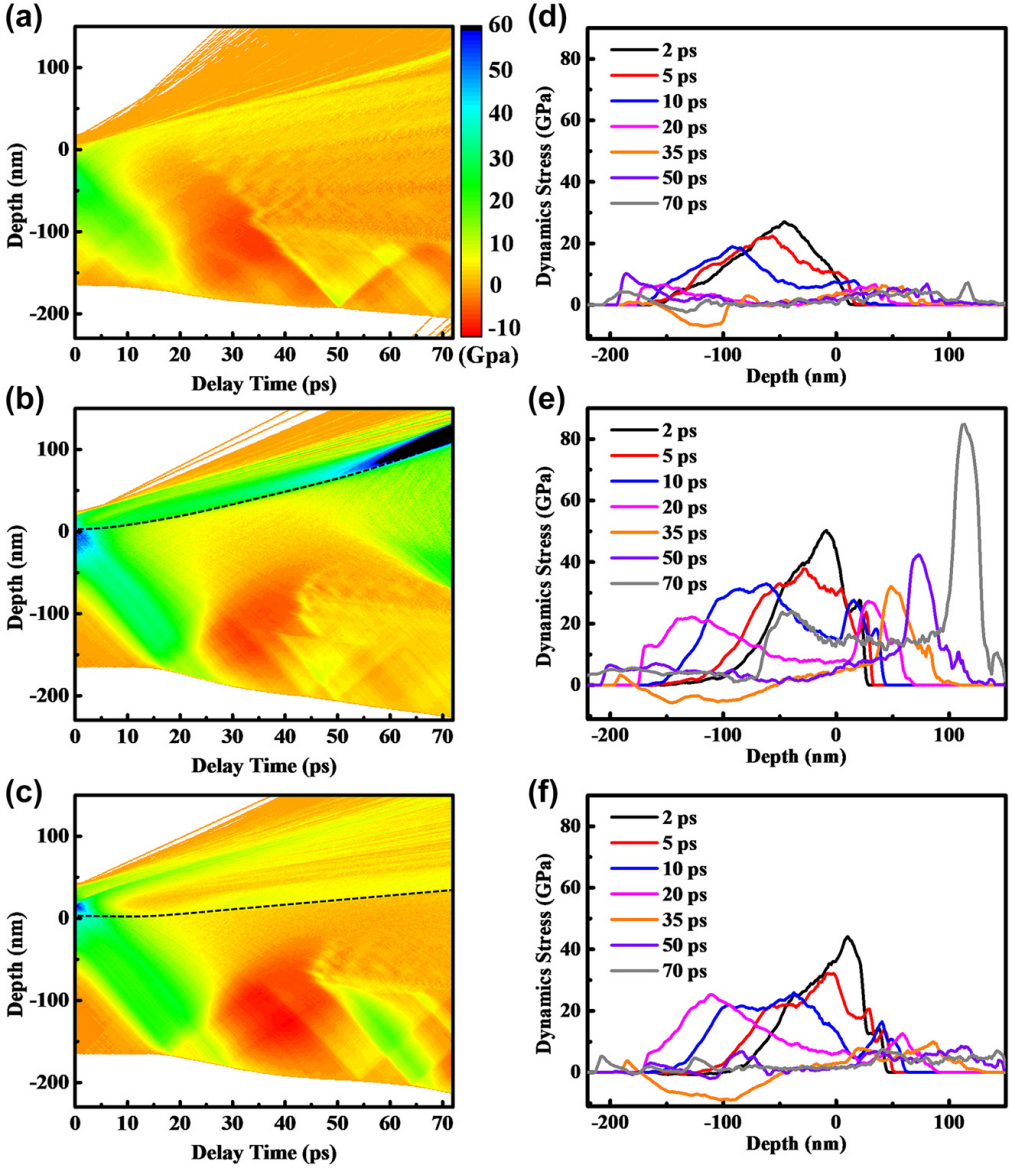
The spatio-temporal evolution of dynamic stress is depicted in Panels (a), (b), and (c), which show stress contour maps for the single-layer Ti, 20 nm Au covered Ti, and 35 nm Au covered Ti samples. These patterns illustrate the evolution of stress with depth and time within 70 ps. Panels (d), (e), and (f) depict stress profiles at specific time delays corresponding to Panels 7 (a), (b), and (c), respectively. The black dotted lines indicate the Au–Ti interface.

In Figure 7(a), the single-layer Ti film exhibits vaporization on the surface and the formation of a compressive stress region beneath the surface. This compressive stress propagates deeper over time, reaching the bottom at around 20 ps. At the atomic level, the stress wave compresses the lattice, resulting in a decrease in interatomic spacing and a reduction in the energy carried by the wave. As a result, the peak stress decreases. Between 20 ps and 50 ps, a notable tensile stress area emerges due to the reaction force. However, at longer delays, there is no prominent stress peak, and the maximum instantaneous tensile stress in the remaining Ti film is below 5 GPa. In Figure 7(b), in the case of the 20 nm Au-covered sample, the substantial electron-lattice coupling coefficient of the underlying Ti leads to a rapid rise in the surface temperature of Ti. This leads to a rapid transition from solid to liquid and then to vaporization of Ti. As a result, a compressive stress is generated with the highest peak value and the longest duration, propagating through the depth of the thin film. At 50 ps, a compressive stress exceeding 40 GPa is generated in the sub-layer of the Au film that has not completely melted. With increasing delay, the compressive stress increases to over 80 GPa. At 70 ps, a secondary inwardly transmitted compressive stress wave emerges at a depth of 80 nm, maintaining a compressive stress of over 25 GPa. Even with an increased thickness of 35 nm for the Au film in Figure 7(c), a second compressive stress wave still appears. However, in Figure 7(c), both the maximum stress magnitude and the duration of the second compressive stress wave are lower when compared to Figure 7(b). This suggests a diminished mechanical response in Figure 7(c).

**Figure 8:**
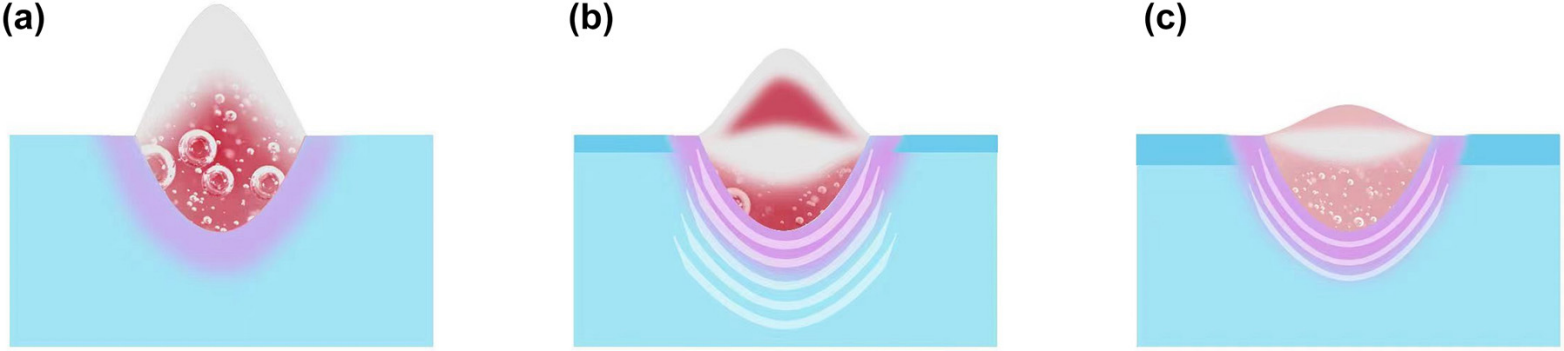
Ultrafast response and ablation mechanisms of (a) single-layer Ti, (b) 20 nm-covered Ti, and (c) 35 nm-covered Ti.

Figure 8 illustrates the ultrafast response and ablation mechanisms of Au–Ti systems under femtosecond laser irradiation. In the case of the single-layer Ti film depicted in Figure 8(a), the energy from the electron system is primarily transferred to the surface lattice through electron-phonon coupling. As a result, there is a swift increase in surface temperature, leading to the melting and vaporization of the material. This results in the formation of a Gaussian-shaped ejection front comprising vapor-phase particles that are expelled at high velocities. Concurrently, the liquid Ti expands outward, forming a three-layer structure comprising vapor, liquid, and solid phases (Figure 5(a)). Due to the absence of a second reflective surface for interference and the extremely rapid ejection, Newton’s rings cannot be observed (Figure 1(a)). This suggests that the removal of the Ti film is mainly driven by thermal effects. When the surface is heated, an instantaneous thermal stress is generated, compressing the underlying lattice and triggering an inwardly propagating compressive stress wave. As the compressive stress relaxes and a tensile stress wave is released outward, a series of cavities form and gradually merge, ultimately resulting in the formation of micro-pits in the irradiated region (Figure 3(a)). By the time the tensile stress reaches the surface, the previously high-temperature liquid phase has already evaporated, leading to the dissipation of tensile stress from the inside out. As a result, no distinct second compressive stress wave is observed in the simulation results (Figure 7(a)), and the peak stress value is the smallest among the studied cases (Figure 7(d)).

In Figure 8(b), we observe the removal mechanism when the Ti film is covered by a 20 nm layer of Au. The Au film increases the overall reflectivity and absorbs a small portion of the energy. The Au surface undergoes melting and vaporization but at a slower rate compared to Ti due to its lower heating rate. As a result, the Au remains in a low potential energy state, coexisting between solid and liquid phases, while the Ti in the vapor phase expands (Figure 6(b)). The residual Au suppresses the expansion of the vapor-phase Ti, resulting in high vapor pressure (Figure 7(b)). As the expansion progresses, heat transfer from the vapor-phase Ti raises the potential energy of the Au surface, resulting in melting. In this situation, both the Au surface, vapor-phase Ti, and residual Ti surface meet the criteria for enhancing interference, as demonstrated in Figure 1(a). This validates the precision of the theoretical simulations. From a mechanical perspective, after the initial formation of the thermal stress wave, the suppressed vapor-phase Ti generates a significant inward compressive stress wave with a strength of tens of gigapascals (Figure 7(b)). This causes severe lattice damage in Ti, ultimately resulting in a substantial enhancement of ablation (Figure 3(b)). In Figure 8(c), when the Au film thickness is increased to 35 nm, more energy is deposited in Au and less in Ti. As a result, the phase transition in Au becomes more pronounced, while the phase transition in Ti slows down (Figure 6(c)). As a result, the impact of Ti on Au lessens, resulting in a slower ejection process and a reduction in the spacing of Newton’s rings (Figure 1(a)). The inward pressure generated by the vapor-phase Ti also decreases, leading to a reduction in the magnitude of the second compressive stress wave (Figure 6(c)). Consequently, Ti is not fully removed, and only micro-cracks form on the surface of the remaining Ti.

## 4 Conclusions

By integrating experimental investigations and theoretical simulations, we have examined the energy transfer and ablation processes in Au–Ti bilayer systems. The photon energy deposition, interface thermal-mechanical coupling process, and damage mechanisms have been revealed.

(1) The Au film reduces the energy deposition in the underlying Ti layer and lowers the phase transition strength of Ti. Because of the higher electron-lattice coupling rate, Ti undergoes a more rapid temperature increase. This leads to the formation of vapor-phase Ti, which subsequently pushes the surface Au layer outward, while the Au layer remains in a combined solid-liquid state.
(2) The sandwich phase structure involving residual Ti – vapor Ti – erupted Au is confirmed by Newton’s rings in pump probe images and MD-TTM simulations. The erupted Au layer hinders the expansion of vapor-phase Ti and forms dynamic stress that compresses the underlying residual Ti layer. As compressive stress transforms into tensile stress, it will cause mechanical damage to the material.
(3) Increased Au thickness decreases the melting and evaporation rate but enhances the mechanical response. Therefore, the strongest mechanical response forms with 20 nm Au-covered Ti. Both Au and Ti are completely removed.

It is important to note that the materials investigated in this study, Au and Ti, exhibit high electrical conductivity and strength, respectively. Their combination as electrodes finds widespread applications in areas such as capacitors, micro-electro-mechanical systems (MEMS), and optoelectronic sensors. The findings presented in this paper can offer valuable insights for the design of Au–Ti composite systems, which is critical for achieving efficient and low-loss processing, thus greatly enhancing the functional capabilities of corresponding devices. Furthermore, the utilization of ultrafast pump-probe imaging in conjunction with MD-TTM modeling, as demonstrated in this paper, shows significant potential in the examination of ablation processes in heterogeneous materials. This approach holds promise for application in various material systems in future research.

## Supplementary Material

Supplementary Material Details

Supplementary Material Details
