# Supplementary material for: Ultrafast dynamics and ablation mechanism in femtosecond laser irradiated Au/Ti bilayer systems
Source: Nanophotonics. 2023 Nov 30;12(24):4461–73. doi: 10.1515/nanoph-2023-0497 (PMC11501701; doi:10.1515/nanoph-2023-0497)
Supplement: Supplementary file 1 — Supplementary Material Details [file j_nanoph-2023-0497_suppl_001.docx]

**Ultrafast Dynamics and Ablation Mechanism in Femtosecond Laser Irradiated Au/Ti Bilayer Systems**

**Yiling Lian^1^, Lan Jiang^1,2,3^, Jingya Sun^1,2,^*, Wenpan Tao^1^, Zhicheng Chen^1^, Gen Lin^1^, Ziqian Ning^1^, Manlou Ye^1^**

1 Laser Micro/Nano Fabrication Laboratory, School of Mechanical Engineering, Beijing Institute of Technology, Beijing 100081, P.R. China

2 Yangtze Delta Region Academy of Beijing Institute of Technology, Jiaxing 314019, P.R. China

3 Beijing Institute of Technology Chongqing Innovation Center, Chongqing 401120, P.R. China

*****Corresponding author: Jingya Sun

E-mail address: [sjy@bit.edu.cn](mailto:sjy@bit.edu.cn)

**Theoretical modeling of MD-TTM**

The heat transfer between electrons and atoms is implemented by the concept of TTM. It is formulated by combining differential equations describing thermal diffusion within both the electron and lattice subsystems.

| $C_{e}\frac{\partial T_{e}}{\partial t}=\nabla\left[ k_{e}\nabla T_{e} \right]-G\left( T_{e}-T_{l} \right)+S$ | (1a) |
| --- | --- |
| $C_{l}\frac{\partial T_{l}}{\partial t}=\nabla\left[ k_{l}\nabla T_{l} \right]+G\left( T_{e}-T_{l} \right)$ | (1b) |

where $t$ is the time. $T_{e}$ and $T_{l}$ represent the temperatures of electrons and lattice, respectively. $C_{e}$ and $C_{l}$ are the heat capacities of electrons and lattice, respectively. $k_{e}$ and $k_{l}$ denote the thermal conductivities of electrons and lattice, respectively. $G$ is the electron-phonon coupling factor. $S$ is the laser power source. Employment of the MD method in the atom subsystem overcomes the shortcoming of TTM, the lattice temperature $T_{l}$ can be transferred to atom temperature $T_{a}$, which is as follows

| $m_{i}\frac{\partial\vec{v}_{i}}{\partial t}=-\frac{\partial U}{\partial\vec{r}_{i}}+\vec{F}_{i}^{Langevin}\left( T_{e}-T_{a} \right)$ | (2) |
| --- | --- |

where $m_{i}$, $\vec{r}_{i}$, and $\vec{v}_{i}$ is the mass, position vector, and velocity of atom $i$. $U$ represents the potential energy of the atom. The electron-phonon coupled energy transfer is modeled by a force $\vec{F}_{i}^{Langevin}$ from the Langevin thermostat.

The atomistic interactions of the film were computed using the Embedded-Atom Method (EAM) potential. The total energy of atom $i$ is determined through the following calculation:

| $E_{i}=F_{i}\left( \sum_{j\neq i} \rho_{i}\left( r_{i,j} \right) \right)+\frac{1}{2}\sum_{j\neq i} \emptyset(r_{i,j})$ | (9) |
| --- | --- |

The distance between atom $i$ and atom $j$ is$r_{i,j}$. $F_{i}$ which represents the embedding energy of atom i due to interactions with other atoms. This term is a function of $\sum_{j\neq i} \rho_{i}\left( r_{i,j} \right). \sum_{j\neq i} \rho_{i}\left( r_{i,j} \right)$ represents the atomic electron density excluding atom $i$. $\emptyset$ is the pair potential as a function of $r_{i,j}$.

As a result, the time step (∆t) used in the Molecular Dynamics (MD) simulations was set to 1 fs. Additionally, the initial time step for TTM calculations was also chosen as 1 fs, and it dynamically adjusted itself to meet the stability criterion during the simulations. The grid size $\Delta z$ for TTM calculations was the same to $L_{e}$. Therefore, in MD simulations, the grid number in space is 500. Before simulations of femtosecond laser irradiation, the MD model was relaxed at $300 K$ and $1 bar$ under the NPT ensemble for $70 ps$. The simulation of the phase change induced by the femtosecond laser was conducted within a time interval of $t_{sum}=70 ps$ using the NVE ensemble.


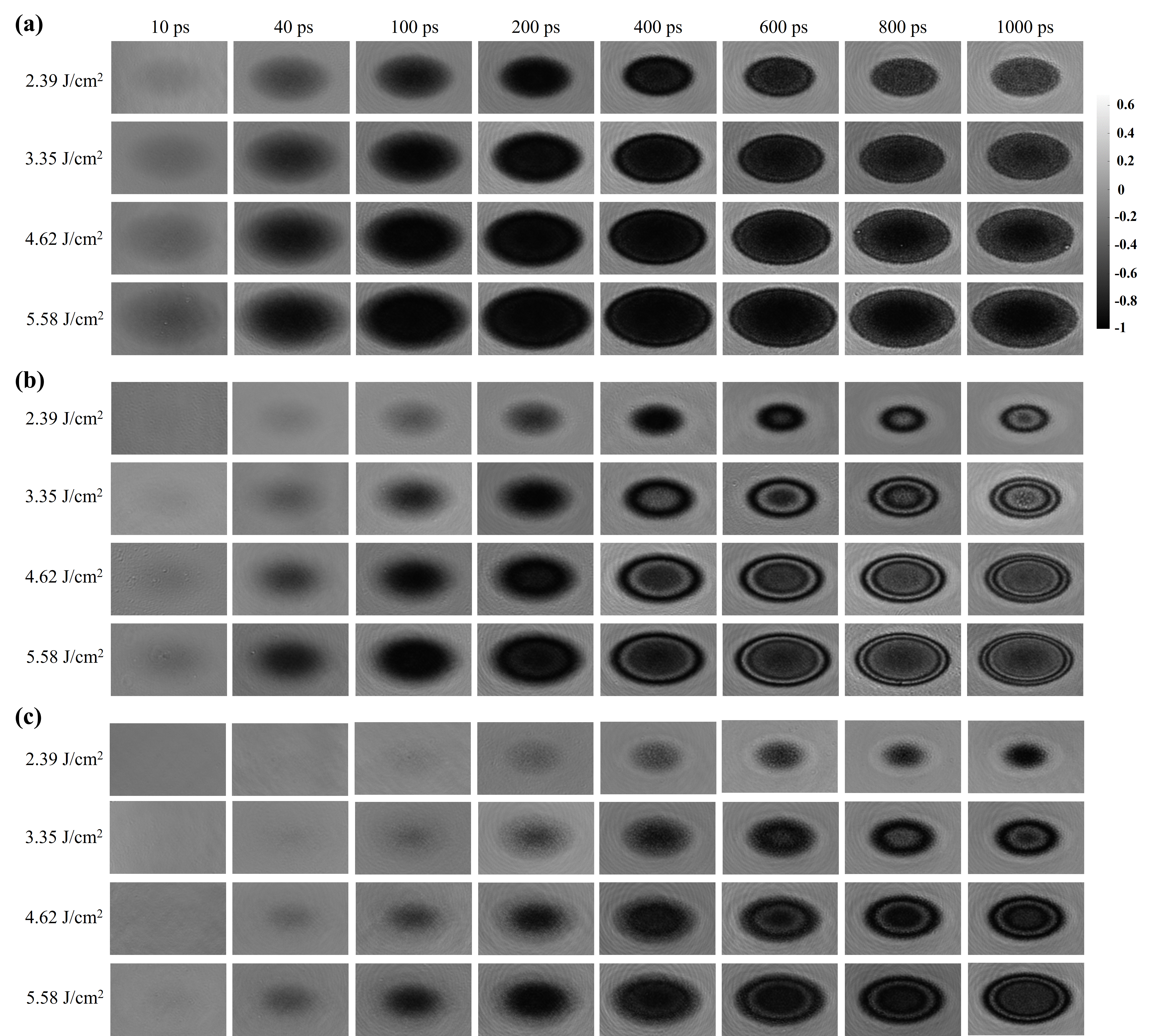


**Figure S1** (a), (b) and (c) are the original time-resolved reflective pump-probe images corresponding to Figure 1 (b), (c) and (d).
